# Early detection of mild cognitive impairment through neuropsychological tests in population screenings: a decision support system integrating ontologies and machine learning

**DOI:** 10.3389/fninf.2024.1378281

**Published:** 2024-10-16

**Authors:** Alba Gómez-Valadés, Rafael Martínez-Tomás, Sara García-Herranz, Atle Bjørnerud, Mariano Rincón

**Affiliations:** ^1^Department of Artificial Intelligence, Universidad Nacional de Educación a Distancia (UNED), Madrid, Spain; ^2^Cogni-UNED Research Group, Faculty of Psychology, UNED, Madrid, Spain; ^3^Computational Radiology and Artificial Intelligence Unit, Department of Physics and Computational Radiology, Clinic for Radiology and Nuclear Medicine, Oslo University Hospital, Oslo, Norway; ^4^Department of Physics, University of Oslo, Oslo, Norway

**Keywords:** ontology, machine learning, SWRL, decision tree, ensemble, decision support system, MCI

## Abstract

Machine learning (ML) methodologies for detecting Mild Cognitive Impairment (MCI) are progressively gaining prevalence to manage the vast volume of processed information. Nevertheless, the black-box nature of ML algorithms and the heterogeneity within the data may result in varied interpretations across distinct studies. To avoid this, in this proposal, we present the design of a decision support system that integrates a machine learning model represented using the Semantic Web Rule Language (SWRL) in an ontology with specialized knowledge in neuropsychological tests, the NIO ontology. The system’s ability to detect MCI subjects was evaluated on a database of 520 neuropsychological assessments conducted in Spanish and compared with other well-established ML methods. Using the *F2* coefficient to minimize false negatives, results indicate that the system performs similarly to other well-established ML methods (*F2_TE2_* = 0.830, only below bagging, *F2_BAG_* = 0.832) while exhibiting other significant attributes such as explanation capability and data standardization to a common framework thanks to the ontological part. On the other hand, the system’s versatility and ease of use were demonstrated with three additional use cases: evaluation of new cases even if the acquisition stage is incomplete (the case records have missing values), incorporation of a new database into the integrated system, and use of the ontology capabilities to relate different domains. This makes it a useful tool to support physicians and neuropsychologists in population-based screenings for early detection of MCI.

## 1 Introduction

Alzheimer’s disease (AD) is the most common cause of dementia affecting the elderly (Jitsuishi and Yamaguchi, 2022; Sherimon et al., 2021; Zekri et al., 2015), and its incidence is expected to continue to increase as the population ages (Ivascu et al., 2015; Zhang et al., 2014). Mild Cognitive Impairment (MCI) has attracted a great deal of attention as a transitional stage between normal aging and AD (Jitsuishi and Yamaguchi, 2022; Panza et al., 2005; Zhang et al., 2014). Early detection of this stage is of vital importance for appropriate early intervention to help slow disease progression and improve patients’ quality of life (Ivascu et al., 2015; König et al., 2018). Therefore, significant efforts have been dedicated to identifying more efficiently early features and symptoms of MCI (Petersen et al., 2014) which has produced an exponential growth of biomedical data (Hoehndorf et al., 2015).

In recent years, ML techniques have been used to obtain an early diagnosis of MCI, either using MRI imaging (Jitsuishi and Yamaguchi, 2022) or neuropsychological tests (Clark et al., 2016; Linz et al., 2017; López-de-Ipiña et al., 2018) due to their capability of handling large amounts of information and obtaining clinically relevant knowledge (Sherimon et al., 2021; Weakley et al., 2015). But for this information to be useful, and the results obtained in studies with ML models to be generalizable, the data must be in a standardized format (Gomez-Valades et al., 2021; Sherimon et al., 2021; Zhang et al., 2014). This allows efficient retrieval of data (Patrick and Li, 2012; Sahoo et al., 2022), shareability between different centers (Gomez-Valadés et al., 2019), and univocal interpretation (Sherimon et al., 2021). Otherwise, the analyses could lead to different interpretations at centers other than where the data came from, or even in the same center because inexperienced staff may not be familiar with the original guidelines, or the population distribution changes over time. This is extremely critical in the healthcare field (Sherimon et al., 2021).

In this scenario, ontologies play a critical role in the management and interoperability of information, allowing the consistent representation of knowledge, standardizing data acquired and stored under different formats and protocols (Costa, 2014; Hoehndorf et al., 2015), providing a unique meaning to each element (Sherimon et al., 2021), avoiding interoperability problems (Gomez-Valadés et al., 2019; Kulmanov et al., 2020), easing the retrieval of information and records (Patrick and Li, 2012), and improving data analysis and efficiency of clinical diagnostic support systems (Shoaip et al., 2019).

Thus, on the one hand, ML models are used to obtain knowledge by searching for patterns of interest in large volumes of data (Tsymbal et al., 2007; Weakley et al., 2015), while, on the other hand, ontologies provide the basis for reusing and unambiguously integrating domain knowledge within applications (Jensen et al., 2013; Kang et al., 2019; Tsymbal et al., 2007). Our proposal seeks to leverage the benefits of both technologies, which separately have their inconveniences. In the case of ML models, it is usually difficult or even impossible to know the logical process behind a decision (Tsymbal et al., 2007; Weakley et al., 2015). Moreover, as they do not check data integrity, they can operate with conceptually but not technically incorrect data, leading to erroneous patterns when working with poorly curated databases (Sherimon et al., 2021). In the case of ontologies, a high-level representation for the formalization of knowledge (Tsymbal et al., 2007) can reach levels of abstraction and complexity that make their use impractical or not viable in real-world scenarios (Zekri et al., 2015). Although significant efforts have been made to combine both technologies (Kulmanov et al., 2021; Robinson and Haendel, 2020), methods that integrate them into decision support systems are still under development (Kulmanov et al., 2020).

In this paper, we propose to integrate a set of bootstrap aggregated (or bagged) decision trees for early diagnosis of MCI, which are represented as rules using the Semantic Web Rule Language (SWRL), with an already defined Ontology Web Language (OWL) ontology with specialized knowledge in neuropsychological tests, NIO (Gomez-Valades et al., 2021). In this way, the integrated system eases data standardization while providing a fast and interpretable first assessment of the cognitive status of subjects, saving physicians and neuropsychologists time and allowing them to reach a wider population during the screenings.

The rest of the paper continues as follows: Section 2 summarizes the state of the art of other approaches integrating ontologies and ML; Section 3 describes the methodology used to build the integrated system, detailing the ontology, the learning model, and the integration method, as well as the database; Section 4 details the performance results, compares them with other well-established ML models, and presents three use cases that show some advantages of this integration; Section 5 introduces the discussion of these results; and finally, Section 6 closes with the conclusions.

## 2 State of the art

Ontologies and ML models conform the two main technologies for extracting, manipulating, and obtaining new knowledge within a domain (Kulmanov et al., 2020; Sahoo et al., 2022; Tsymbal et al., 2007). It seems logical that proper integration between them would result in an overall improvement in the performance of decision support systems (Kulmanov et al., 2020; Sahoo et al., 2022; Zhang et al., 2014). However, both technologies are usually employed separately (Tsymbal et al., 2007), although recently there has been an increased effort to combine them (Kang et al., 2019). This combination is performed following different objectives, such as the automatic completion of ontologies (Mežnar et al., 2022), the search for emerging knowledge in ontologies using ML techniques (Kulmanov et al., 2021; Robinson and Haendel, 2020), or the improvement of diagnosis in decision support systems.

Within this last group, some studies use ontologies and ML models sequentially: an ontology is first used to standardize and add semantic knowledge to a database, which is subsequently used to train the automatic system (Lakshmi et al., 2019; Sahoo et al., 2022; Tsymbal et al., 2007). Other studies focus on the integration of the predictive ML model in an ontology. Thus, a compact decision support system is generated. In this area, one of the first approaches appears in the work of Zhang et al. (2014) with Ontology-Driven Decision, which combines an ontology with a decision tree to create a decision support system for the early diagnosis of AD employing MRI images. In that system, the ontology is used to standardize the data and reduce subjectivity, while the decision tree generates the diagnosis. To integrate both parts, the decision tree rules were transformed into RDF rules, and the diagnosis was obtained using a reasoner. The work of Shoaip et al. (2021) proposes the integration of an existing ontology, ADDO (Shoaip et al., 2020), with a set of rules extracted from both a decision tree and a Repeated Incremental Pruning to Produce Error Reduction (RIPPER) method (Fürnkranz and Widmer, 1994) to differentiate between four categories (healthy, significant memory concern, early MCI, and late MCI). Unlike the other studies, which use their own database, they use a heterogeneous dataset obtained from the ADNI database (Petersen et al., 2010). This dataset includes neuropsychological tests, imaging tests, and chemical and genetic biomarkers which, together with the sociodemographic variables, are collectively called “biomarkers.” The rules obtained from ML were translated to SWRL rules to integrate them into the ontology. They also link different properties defined in the ontology using SWRL to give rules more expressiveness. Another approach also based on SWRL rules is the one proposed by Massari et al. (2022c) for diabetes detection, which combines a decision tree with ontologies. The diagnosis is obtained through inference using a reasoner. The system described was adapted afterward for the early detection of breast cancer (Massari et al., 2022a) and covid-19 (Massari et al., 2022b).

These papers showed that a proper integration of both, ontologies and ML algorithms, allows heterogeneous data to be accessed and put into a standardized framework, improving the performance of automatic models, and facilitating the exchange of data and results. However, there are still problems that need to be addressed. The first is the need to reuse or adapt previous ontologies in some studies that create their ontologies from scratch. Therefore, they may have redundancies and inconsistencies with prior ontologies. The second one is the selection of a decision tree or a RIPPER as the ML model, which was made to ease its translation and integration to rules (SWRL or RDF), something not possible with more complex ML models due to their black box structure. However, decision trees have strong training set dependency (Zhang et al., 2014), RIPPER methods have problems with noise and complex databases and need categorical data (Kotelnikov and Milov, 2018), and both methods are prone to overfitting if not pruned properly. Shoaip et al. (2021) mention using a decision tree and a RIPPER method to create SWRL rules but need to explain how they combine both sets of rules to function as one. Another problem is that of missing values. The most common ways to deal with missing values are either deleting the affected records or tests or imputing the missing values. However, there is an inherent risk of altering the database and hence the results.

Therefore, in this work we propose a decision support system that integrates a set of decision trees that work together as an ensemble to provide a diagnosis based solely on neuropsychological tests with an already established ontology, NIO. We used neuropsychological tests for being cheaper, faster, and less invasive than the alternatives while keeping a good diagnostic capability, making them the most suitable for population screenings. The ontological part will allow the data to be standardized and placed in a semantic context, and the tree ensemble will establish a diagnosis that combines the explainability of decision trees with the power and robustness of an ensemble method. The system is also designed to operate directly with databases with missing values without the need for prior preprocessing by deleting or imputing records.

## 3 Materials and methods

### 3.1 System modeling

**Figure 1:**
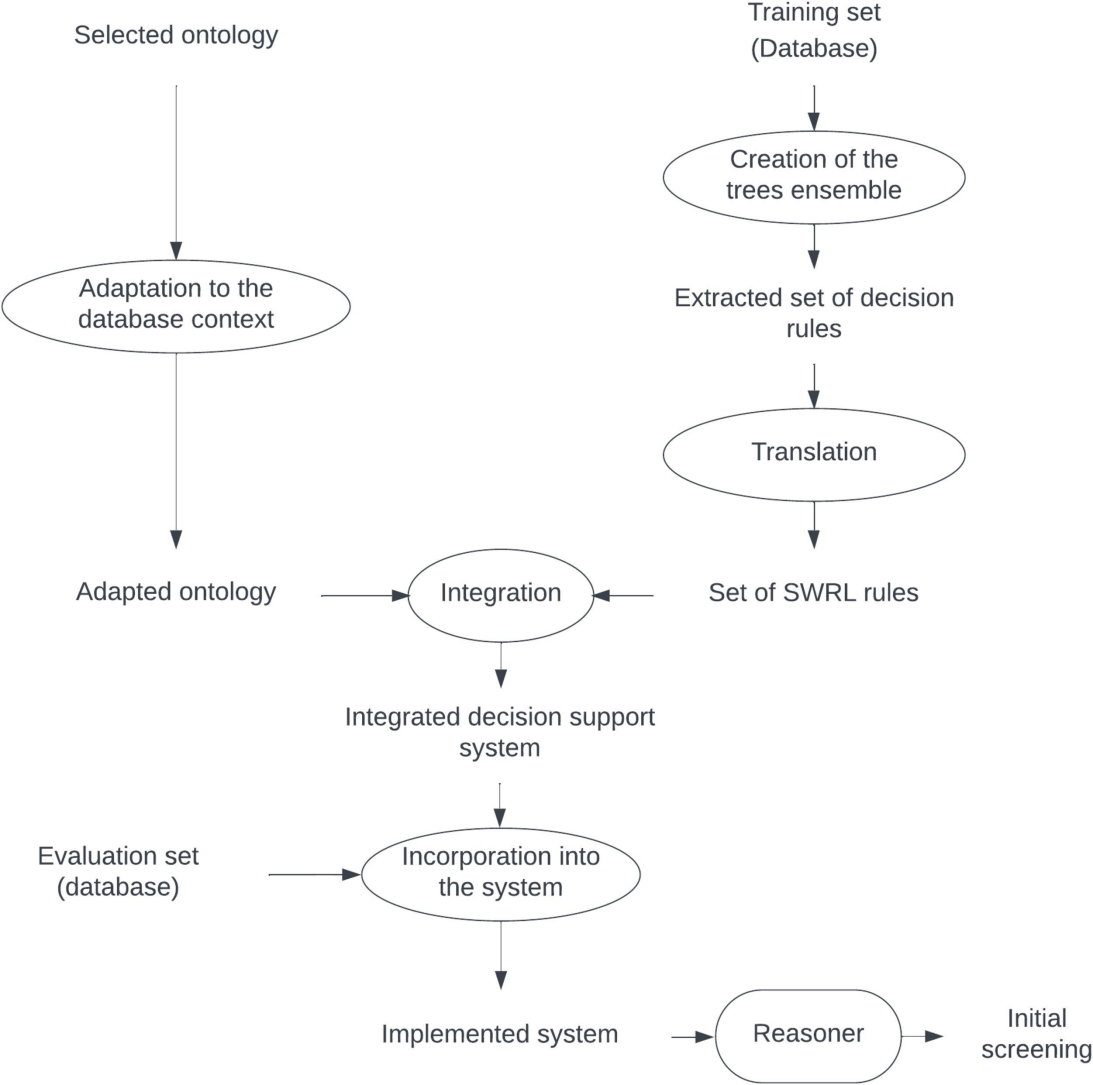
Scheme of the creation of the integrated support decision system between the ontology and ML.

To generate the decision support system that supports the diagnosis, the process was split into three stages, as shown in the diagram in Figure 1: (1) ontology selection and adaptation, (2) generation of the ML model (tree ensemble), translation to SWRL rules and integration within the ontology, and (3) the database is loaded into the ontology, and a reasoner compatible with the SWRL rules is used to infer the diagnosis. Note that in this approach the set of rules is particularized for the specific neurological test battery used in that dataset. Within the decision support system, different sets of rules could coexist, one for each test battery. The results aggregation rule that establishes the final diagnosis only uses the trees associated with a particular neuropsychological test battery, excluding any other decision trees that might be present. The following sections explain the detailed process followed in each stage.

#### 3.1.1 Database

To show how the integrated system works, we used an anonymized database formed by a sample from a large longitudinal study on the incidence of incipient MCI in the Autonomous Community of Madrid (Spain; Díaz-Mardomingo et al., 2017; Díaz-Mardomingo and Peraita, 2008; García-Herranz et al., 2016, 2019; Peraita et al., 2011). Subjects with a previous diagnosis of neurodegenerative disease, disabling chronic disease, psychiatric disorders such as major depression, established neurological abnormality, severe sensory impairment, diabetes, stroke, or loss of consciousness were excluded from the database. The cognitive and emotional status of the subjects was assessed using the Spanish version of the Mini-Mental State Examination (Lobo et al., 1979) and the Geriatric Depression Scale (Yesavage et al., 1982). The diagnosis of MCI was established based on the Petersen criteria, considering tests that evaluated different cognitive abilities (García-Herranz et al., 2016). The study gathered data from 233 monolingual Spanish subjects aged between 58 and 93 years and with an educational level between 0 and 22 years of study. Each subject underwent from one to three evaluations, spaced approximately 1 year apart, classified on each one as Healthy or MCI. This process yielded a total of 520 cases, which we considered as independent in this study to make the most of the small sample. Table 1 shows the summary of the sociodemographic variables in the database.

**Table 1:** Summary of the sociodemographic variables of the database, as well as their performance on the MEC (Spanish version of the MMSE).

Classification	No. of subjects	Men/Women	Age mean (std.)	Scholarity mean (st)	MEC mean (std)
Healthy	309	80/229	70.69 (6.03)	11.64 (5.18)	32.94 (2.06)
MCI	211	59/152	73.00 (6.96)	9.19 (6.06)	30.69 (3.12)

#### 3.1.2 Ontology and rule system

The NIO ontology (Gomez-Valades et al., 2021) was selected for this project because it includes many neuropsychological tests. As NIO is a large ontology with many classes and axioms, it was analyzed and reduced to the appropriate *Classes* for the study to ease its handling and prevent its size from slowing down the reasoner. We used SWRL to integrate the ML model into the ontology since it allows writing rules for reasoning and inferring new knowledge in OWL. We select the SWRL because, since it is rule-oriented, is possible to translate certain machine learning systems such as decision trees as a rule set, that can be integrated inside an ontology. Finally, to enable the rules to function with the data, the tests were defined as *Individuals*, and the scores and sociodemographic variables as *Data Properties* associated with these *Individuals*.

#### 3.1.3 ML model

Decision trees emerge as the most suitable ML models because their rules can be expressed as a concatenation of conditionals. This allows easy translation to other types of rule systems and simple inference interpretation. However, decision trees are unstable and prone to overfitting, which could lead to inaccuracies and make them less competent for complex problems (Ho, 1995). An improvement is bagging, an ensemble learning method based on a set of bootstrap aggregated decision trees whose combined classification is more robust and accurate than the individual decision trees that comprise it and is commonly used to reduce variance.

To train the model, we used 80% of the dataset for training and 20% for testing. From the training set, various sampling subsets of the same size (25% of the training set) were obtained by sampling with replacement to train different decision trees. To keep the explainability of the bootstrap, a reduced number of decision trees was defined. The final classification was obtained by voting, using an odd number of decision trees to avoid ties. The threshold was defined as the minimum number of trees that maximized recall without falling into a trivial classification.

Once the model was obtained, it was translated into SWRL rules. The SWRL rule system is monotonic, which has the following implications:

- The system uses deductive reasoning.
- Rules always move from antecedent to consequent.
- A rule is only activated if all parts of the antecedent are true, so the consequent is also true.
- The system uses valid and known elements, not incomplete or unknown facts.
- The results are always true, so there is no possibility of modification or retraction. Therefore, the addition of new knowledge does not modify the previous knowledge of the model, unlike non-monotonic systems, which can change according to situations or conditions consistent with new knowledge.

Taking that into account, to translate each decision tree to SWRL, each leaf of the tree was converted into a SWRL rule (Massari et al., 2022a; Shoaip et al., 2021). Here is an example:

The leaf of the decision tree:

*“if (Praxias_cons < = 9.5) and (cal_rey < = 24.5) and (TrailATi > 32.5) and (cal_rey > 14.75) then class: MCI (proba: 81.16%).”*

was translated to:

*“Subject(?p) ^ has_praxias_score (?p, ?PC) ^ swrlb:lessThanOrEqual (?PC, 9.5) ^ has_Rey_complex_figure_score (?p, ?CR) ^ swrlb:lessThanOrEqual (?CR, 24.5) ^ has_Trail_Making_test_A_score (?p, ?TMA) ^ swrlb:greaterThan (?TMA, 32.5) ^ has_Rey_complex_figure_score (?p, ?CR2) ^ swrlb:greaterThan (?CR2, 14.75) - > pred_n(?p, 1).”*

To aggregate the decisions of different trees and give the final diagnosis, it is not possible to use the “count” operation because it is not supported in SWRL (the variable “count” would have to change every time an increment occurs and, according to implication e) of the monotonic systems defined above, this is not allowed. Instead, the final classification is established through the “sum” of each tree prediction, which should always be numerical (e.g., 0 for Healthy and 1 for MCI). The rule that adds up the individual classifications of each tree is always executed after all trees have issued a decision.

To identify the optimal threshold of the system, both the *ROC* curve and the *Precision-Recall (P-R)* curve were analyzed. To detect the threshold that maximizes the system sensitivity to the target Class (MCI), the *F-score* curve was analyzed for different values of *β*. The *F-score* is a relation between *precision* and *recall* in which, depending on the value of *β*, both metrics contribute equally to the score (*β* = 1) or more importance is given to *precision* (*β* < 1) or to *recall* (*β* > 1).

#### 3.1.4 System implementation

We used the following environments to implement the system: Python 3.4 with the Scikit-Learn (Pedregosa et al., 2011) module was used to generate the decision trees and their automatic translation into SWRL rules; Protégé 5.6.1 (Musen, 2015) for ontology management due to its ease and wide use; the Protégé SWRL Tab plugin 2.1.0 (O’Connor et al., 2005) for the incorporation and management of SWRL rules; the Cellfie plugin to load the database into the ontology; and Pellet (Sirin et al., 2007) as the reasoner to establish inferences since it is capable of operating with SWRL rules.

## 4 Results

The reliability of the integrated system in identifying cases with MCI was assessed by evaluating its performance in accurately classifying the cases. The versatility and ease of use of the system were also demonstrated through three additional practical use cases based on real-world scenarios: screening of new cases with the possibility of missing records, incorporation of a new database into the system, and the use of ontological capabilities to link different domains and generate new knowledge.

### 4.1 Tree ensemble performance

**Figure 2:**
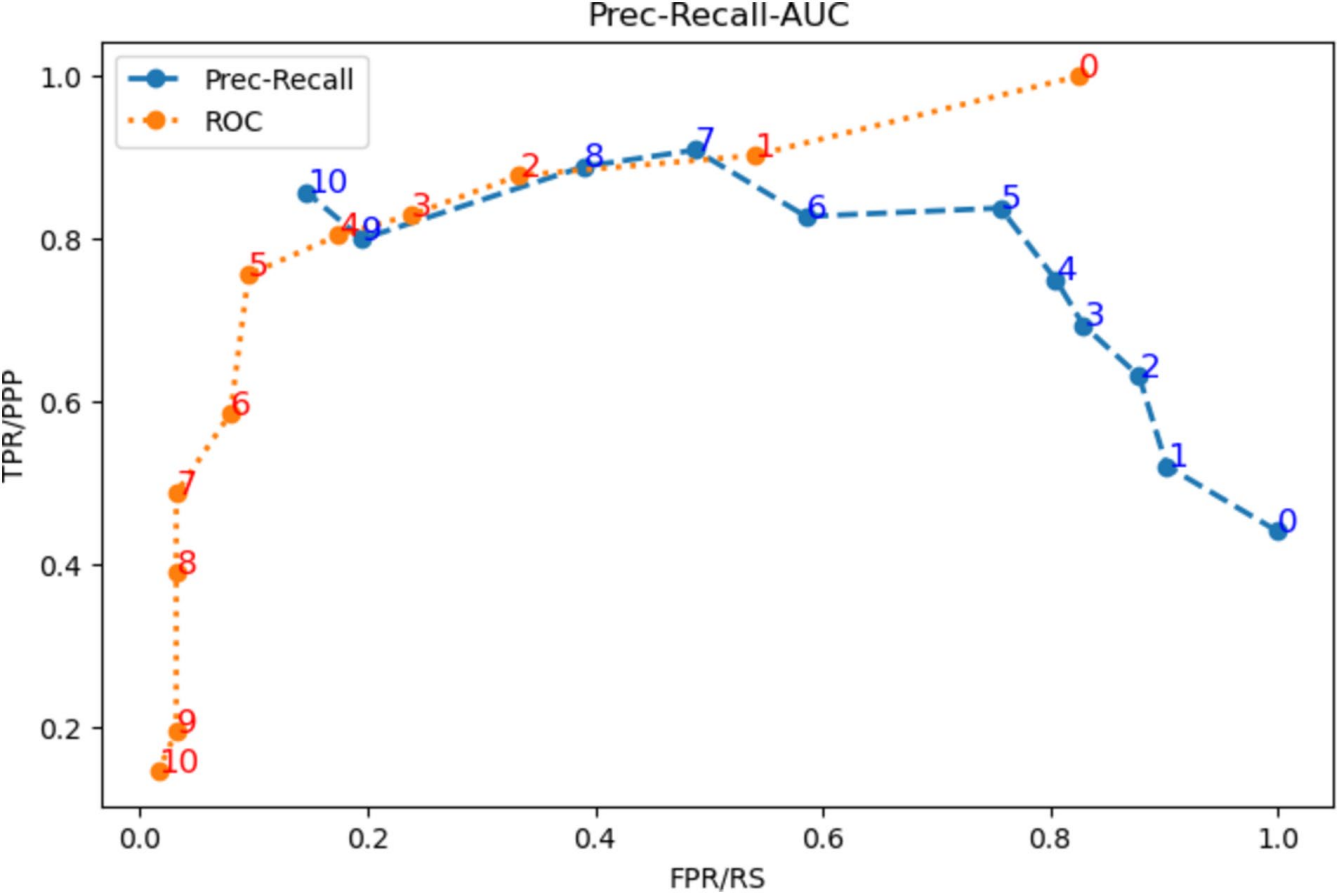
*ROC* curve (orange) and *P-R* curve (blue) for all possible thresholds of the system.

**Figure 3:**
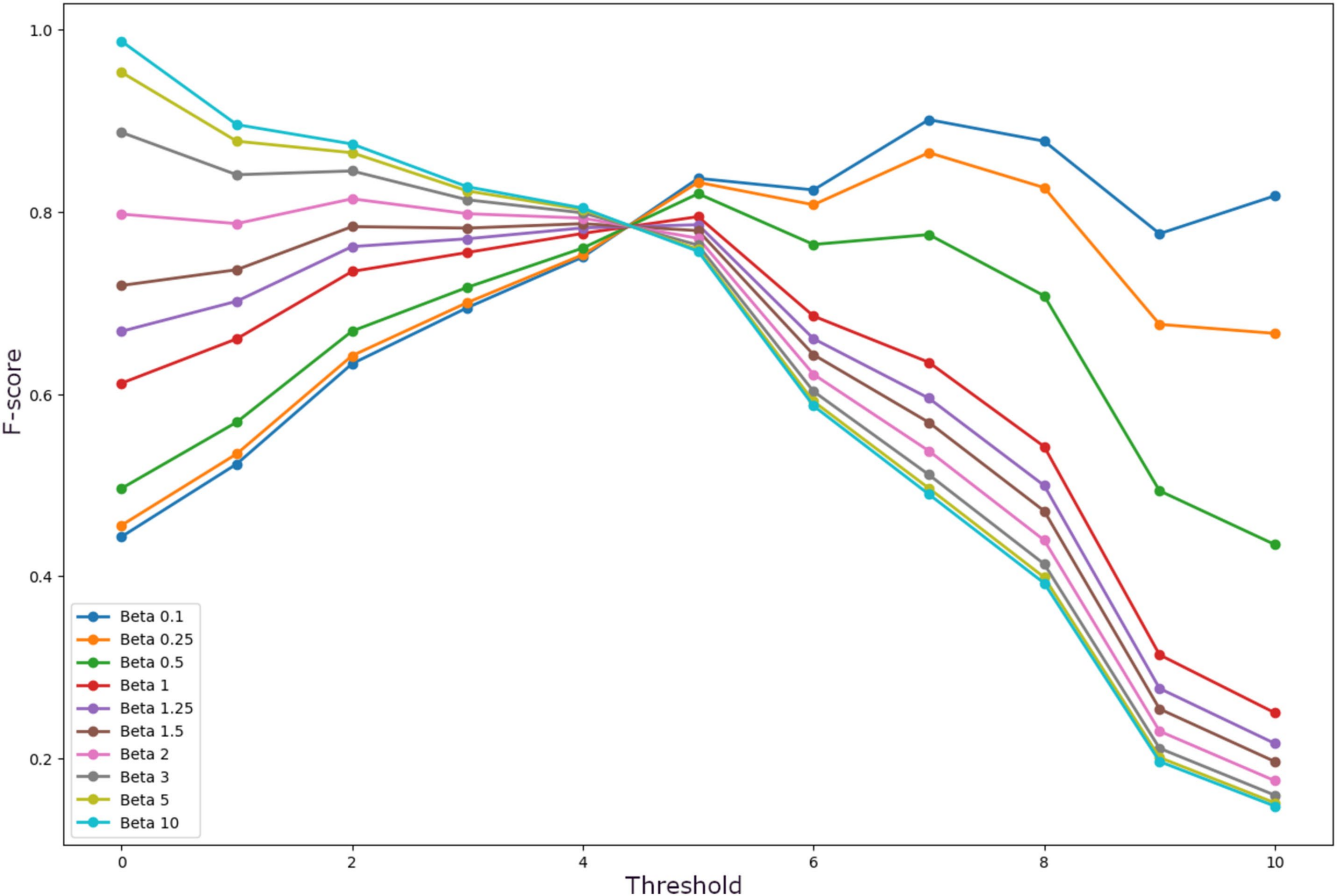
F-score curves for different B for all possible thresholds of the system.

Establishing a rule-based decision system allows fast and direct modification of the threshold to suit it to the context of the study (initially, screening). First, both the *ROC* and the *Precision-Recall* curves were used to identify the optimal threshold. As it is shown in Figure 2, the inflection point in both curves is at threshold 5 (*th* = 5). Next, the behavior of the *F-score* curves was analyzed to establish the threshold to optimize the *recall*, i.e., the minimum number of decision trees necessary to classify a sample as MCI that maximizes *recall* while keeping *precision* high. These curves are shown in Figure 3. Excluding the trivial option of *th* = 0, for all curves with *β* > 1, an inflection is observed at *th* = 2. It is also shown that majority voting leads to lower *F-scores* for all *β* > 1, ratifying the results obtained in the analysis of the *ROC* and the *P-R* curves.

Different numbers of decision trees were evaluated, and the number of 11 decision trees was selected for our system since it obtained the best performance while maintaining a manageable number of trees. Table 2 shows the comparison between the average performance of the 11 trees independently and the tree ensemble for three different thresholds: the majority voting option (*th* = 6), the overall most efficient threshold obtained by the *ROC* and *P-R* curves (*th* = 5), and the best threshold to reduce *false negatives* (*th* = 2). It demonstrated that there is an overall improvement in the tree ensemble concerning the average performance of the individual decision trees. Between the three thresholds, *th* = 6 is outperformed by both *th* = 5 and *th* = 2 in all metrics except *precision*. The comparison of system performance between *th* = 5 and *th* = 2 yields better results for *th* = 2 in *F2* and *recall*, while *th* = 5 presents better results in *accuracy, precision*, and *ROC-AUC*, as expected. However, *F1* remains the same for both thresholds. Using *F2* as the discriminant metric, the tree ensemble (TE) with *th* = 2 was selected as the most appropriate for evaluating performance (*F2_TE2_* = 0.830), widely surpassing both th = 6 (*F2_TE6_* = 0.691) and *th* = 5 (*F2_TE5_* = 0.730).

**Table 2:** Comparison of the performance of the individual decision trees concerning the ensemble using the thresholds corresponding to majority vote (*th* = 6), *ROC/PR* curve (*th* = 5), and *Fβ* curve.

Method	F2	Accuracy	F1	Recall	Precision	ROC-AUC
Tree average	0.651	0.746	0.669	0.635	0.721	0.744
Tree ensemble with *th* = 6	0.691	0.817	0.725	0.630	0.875	0.785
Tree ensemble with *th* = 5	0.730	0.820	0.750	0.700	0.826	0.801
Tree ensemble with *th* = 2	0.830	0.775	0.751	0.896	0.654	0.797

### 4.2 Performance comparison with other ML models

Table 3 shows the comparison of the integrated system with *th* = 2 with seven ML models widely used in biomedical data analysis: Adaboosting (ADAB), Bagging (BAG), Multilayer perceptron (MLP), Logistic Regression (RLog), Random Forest (RF), Support Vector Machine (SVM), XGBoosting tree (XGB). Ten repetitions of the analysis were performed with different initialization seeds to ensure the robustness of the results. To allow proper comparisons between all systems, the thresholds for each system were adjusted to optimize *F2.* As can be seen in Table 3, the performance of *F2_TE2_* for the tree ensemble exceeds all the other ML models except for the BAG (*F2_TE2_* = 0.830 vs. *F2_BAG_* = 0.832). Although the difference was expected as both methods are based on the same type of ensemble and the BAG uses a larger number of components, the difference is dim, and our proposal facilitates the explainability of the results and can be used within the ontology without affecting its performance.

**Table 3:** Comparison of a total of 7 ML models: adaboosting (ADAB), bagging (BAG), multilayer perceptron (MLP), logistic regression (RLog), random forest (RF), support vector machine (SVM), XGBoosting tree (XGB) using the thresholds that maximize their performance for *F2*.

Methods	F2	F1	Accuracy	Recall	Precision	ROC-AUC
ADAB *th* = 0.4	0.800	0.628	0.542	0.985	0.464	0.623
BAG *th* = 0.3	0.832	0.772	0.798	0.880	0.711	0.812
MLP *th* = 0.2	0.790	0.694	0.701	0.872	0.578	0.733
RLog *th* = 0.2	0.767	0.644	0.618	0.884	0.552	0.667
RF *th* = 0.4	0.820	0.806	0.845	0.830	0.788	0.844
SVM *th* = 0.3	0.770	0.707	0.734	0.821	0.626	0.751
XGB *th* = 0.4	0.824	0.816	0.852	0.826	0.812	0.851
Tree ensemble with *th* = 2	0.830	0.751	0.775	0.896	0.654	0.797

### 4.3 General system operation

NIO is an ontology with many *Classes*, so it was first reduced to the necessary *Classes* for the study. Additionally, to compare the results of the tree ensemble integrated into the ontology with the original ML model, we added those *Classes* corresponding to the confusion matrix: “TN_Scores” (*true negatives*), “FN_Scores” (*false negatives*), “FP_Scores” (*false positives*) y “TP_Scores” (*true positives*). Finally, all restrictions related to the range of available values for each test were checked to minimize as much as possible the incorporation of database mistakes.

**Figure 4:**
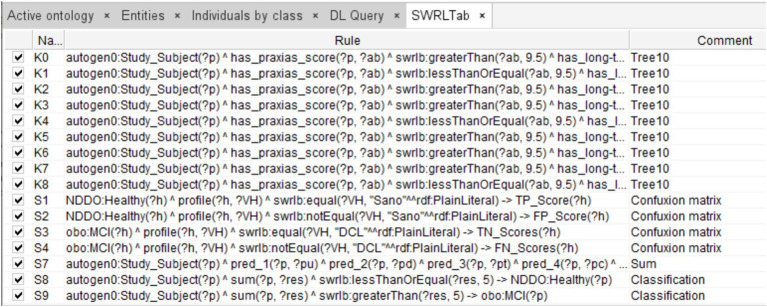
Fragment of SWRL rules corresponding to a complete decision tree (Tree #10), rules for integrating the ensemble predictions, rules of the final diagnosis, and rules defining the confusion matrix (for evaluation purposes).

The rules were integrated into the ontology through the Protégé SWRL plugin, while the database was incorporated through Cellfie. A fragment of this integration that shows the rules corresponding to decision tree #10, the confusion matrix, and the aggregation of the individual tree decisions is shown in Figure 4.

**Figure 5:**
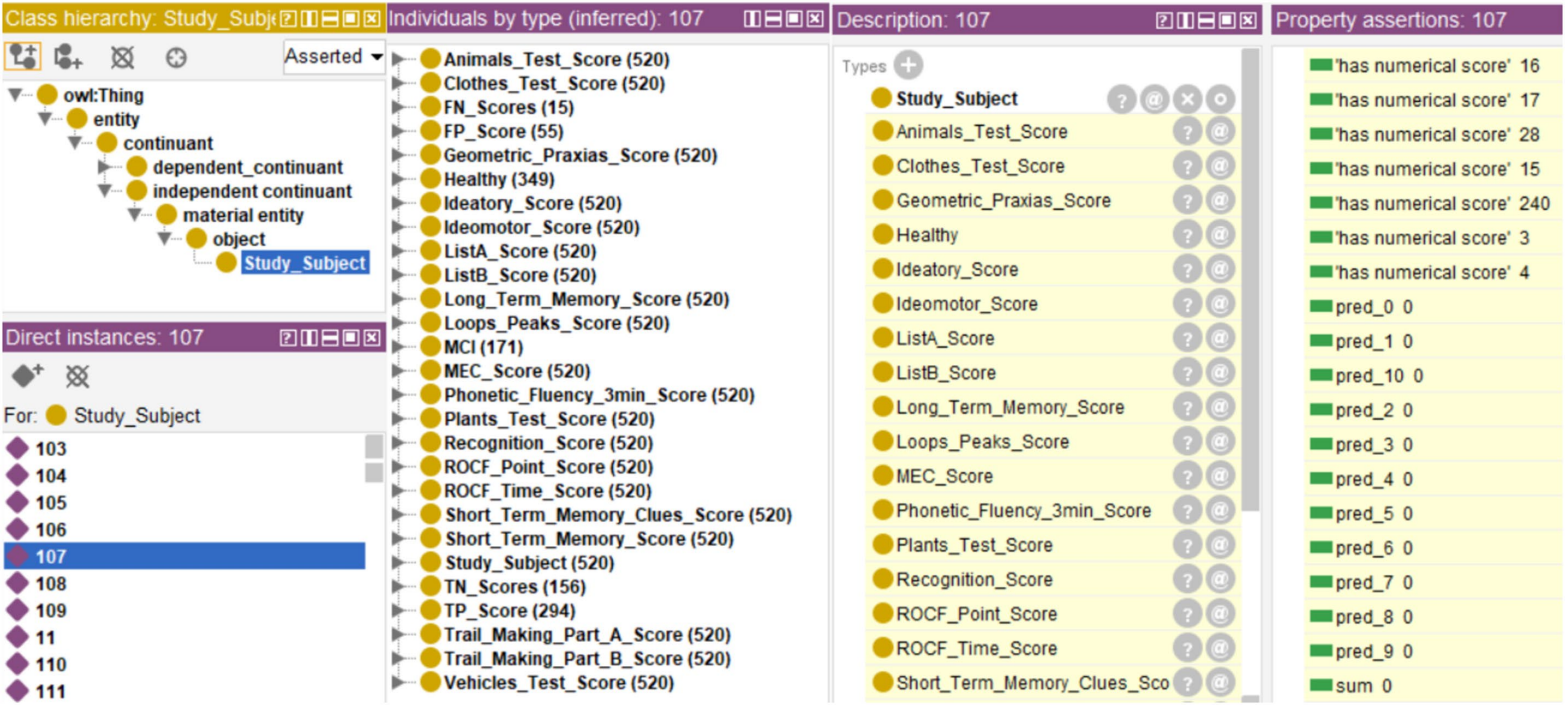
Results inferred by the reasoner, showing in the central window the number of Individuals classified as “Healthy” or “MCI” and the results of the confusion matrix. An example is case 107, showing the prediction per tree (window “Property assertions: 107”) as well as the final diagnosis (inferred in the window “Description: 107”).

The reasoner was activated once the rules and the database were incorporated into the ontology, generating the diagnosis. Figure 5 shows the results inferred by the reasoner for case 107, with “Healthy” and “MCI” corresponding to Healthy and MCI *Classes*, respectively. Each individual tree prediction can be seen under “Property assertions,” where “pred_(*n*)” is a tree, (*n*) is the ID of the tree, and the number following is the classification (0 for Healthy and 1 for MCI). For example, “pred_1 0” means that tree 1 classifies case 107 as “Healthy.” The results of the individual trees are added and stored on the *Data Property* “sum.” In this case, it is 0, and the final classification of that case is “Healthy” since it is less than the threshold (*th* = 2).

**Figure 6:**
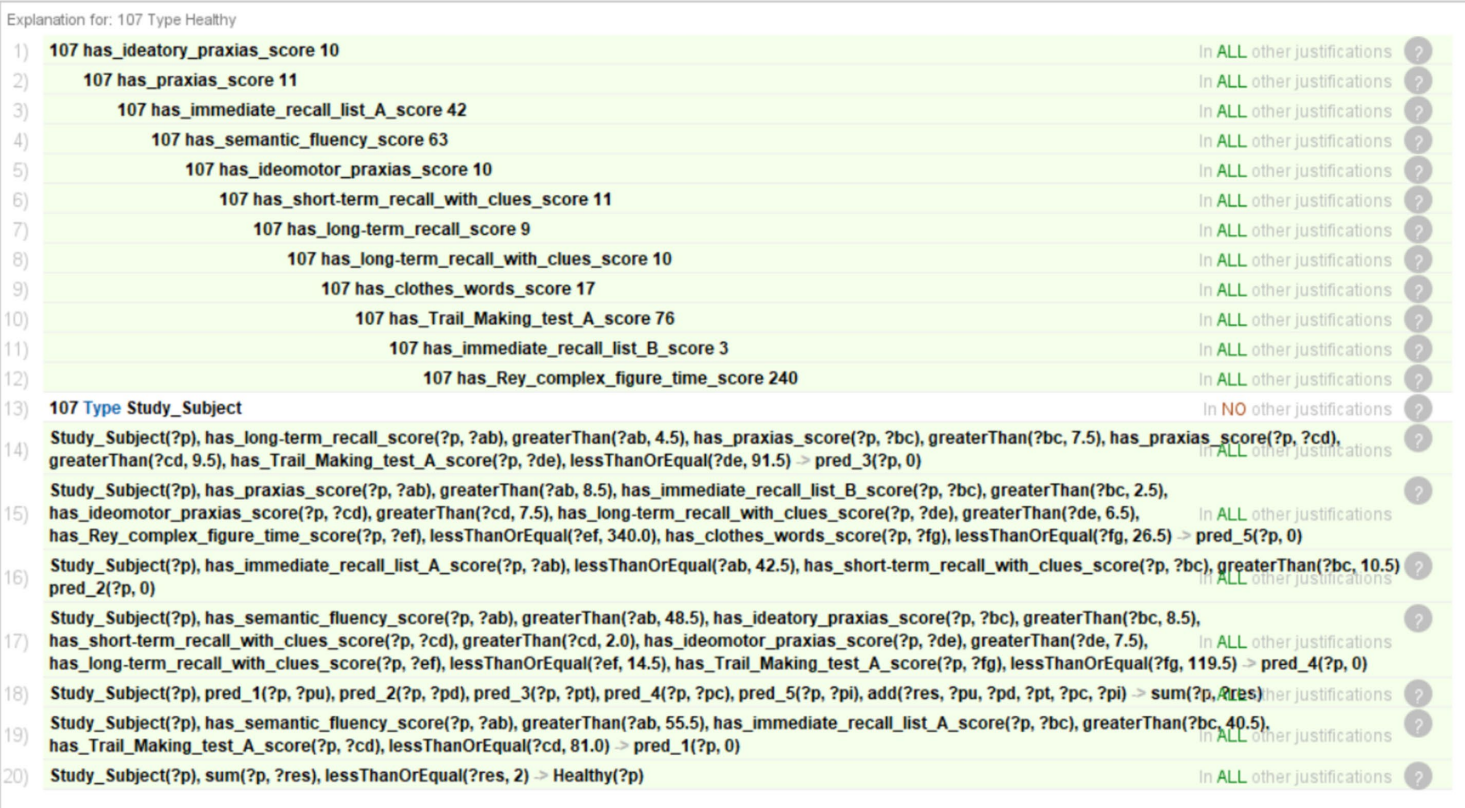
Justification of the reasoning for case 107.

Finally, Figure 6 shows part of the reasoning followed to establish the diagnosis for case 107. This allows the experts to know the process followed by the system to classify a case as “Healthy” or “MCI.”

### 4.4 Other use cases

In addition to the usual classification of individuals belonging to the same population distribution as the sample, the integrated system was evaluated in three other situations to demonstrate its usefulness and versatility:

- To evaluate new cases even when records have missing values.
- To incorporate a new database in the ontology, evaluating similarities and differences between both databases, and making inferences about the new database to obtain a diagnosis.
- To relate different domains and generate emergent knowledge that relates the performance of subjects in certain tests with cognitive domains and associated brain areas already modeled in the ontology.

#### 4.4.1 Screening of new cases

During population screening, a large volume of tests is generated. Those tests need to be evaluated individually, slowing down the screening process. Those tests may lack results in certain parts or subtests due to several factors, such as the refusal or inability of some person to perform a test or a test being applied later in the follow-up. In this case, a decision support system provides an initial classification that can be used to make a first filtering and focusing of the subsequent study. Efficiency increases if the system can handle records with missing data, speeding up the process by avoiding the need to eliminate or preprocess those records beforehand.

**Figure 7:**
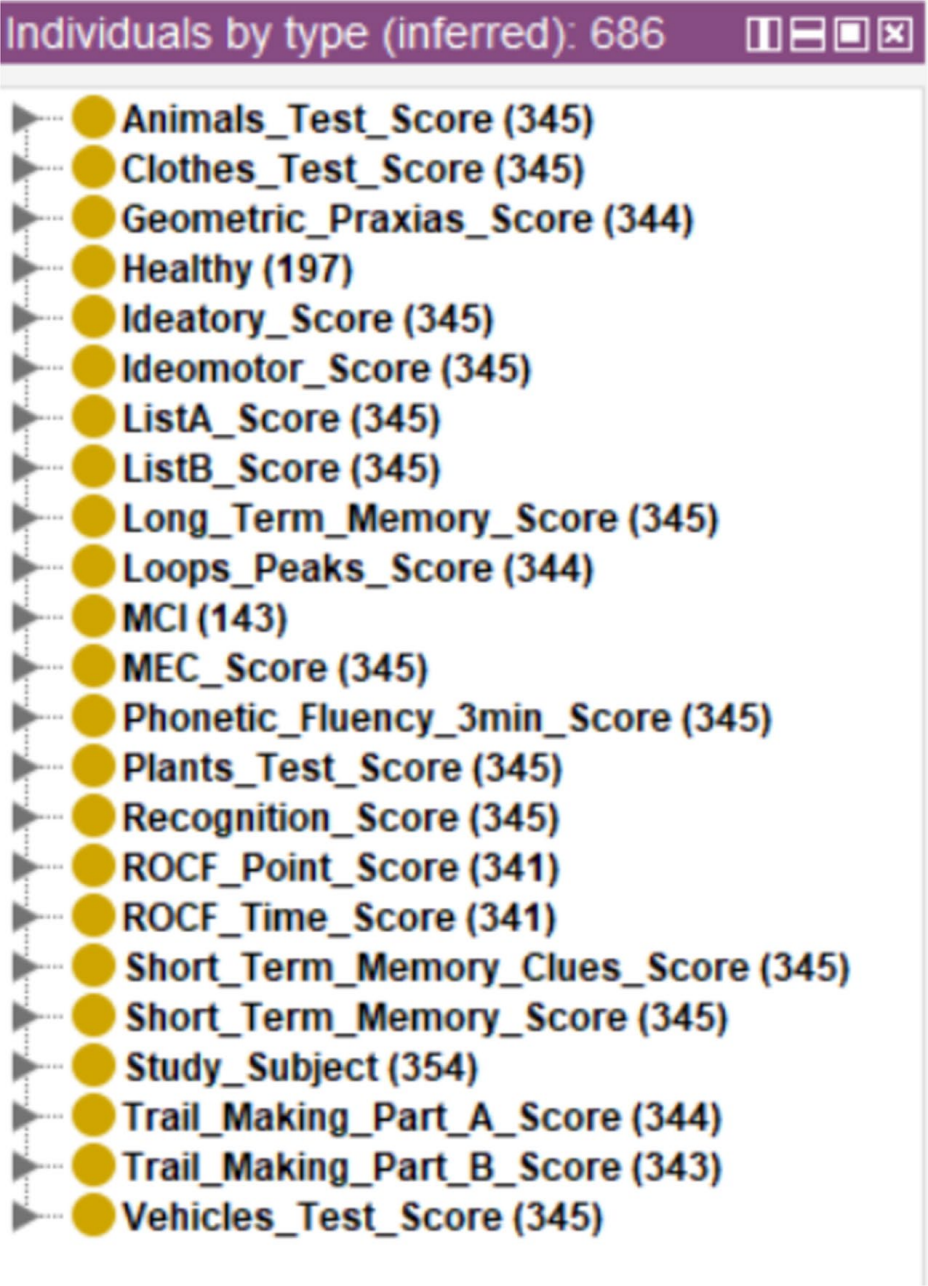
Diagnosis of new cases.

**Figure 8:**
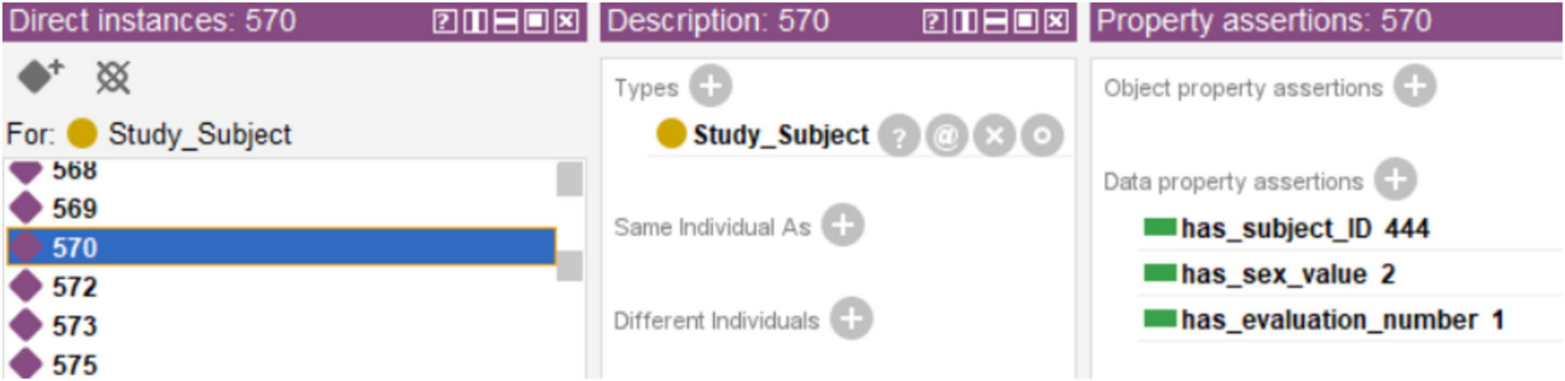
Example of a case with no tests results, showing only the subject ID, the evaluation and the code used for sex.

To exemplify this use case, a new database consisting of 354 cases belonging to the same project but not yet classified will be used. To bring this use case closer to a real screening scenario, this database will be incorporated directly into the system without preprocessing, including those cases with missing records. The data was incorporated into the ontology using Cellfie, running the reasoner next. The threshold used was *th* = 2. The inferred results can be seen in Figure 7. The first thing that can be appreciated is that none of the *Classes* has all 354 cases except the *Class* referring to the test subject identifier (“Study_Subject”). This indicates the presence of records with missing data, as shown in Figure 8 for specific case 570.

**Figure 9:**
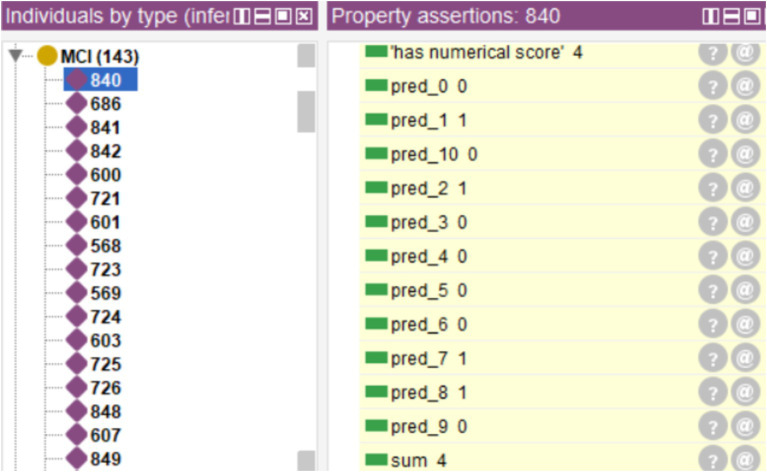
Example of a case diagnosed by the system.

In the cases in which the system issued the final diagnosis, 197 cases were classified as healthy and 143 as MCI, making a total of 340 cases. Figure 9 shows one of these cases, along with the individual diagnoses from the decision trees and the sum used to generate the final diagnosis.

**Figure 10:**
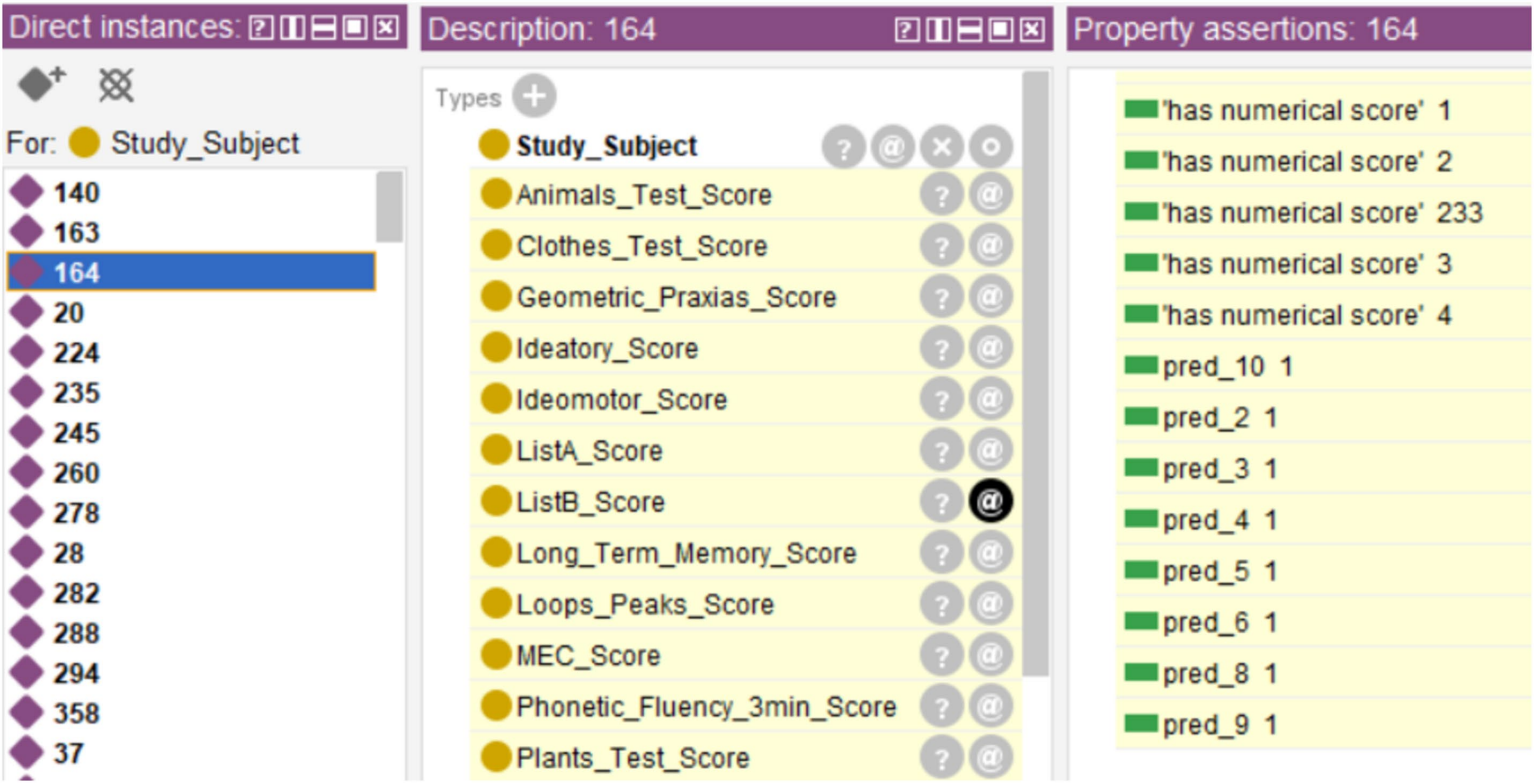
Example of a case with missing values that prevented the generation of the general diagnosis because the diagnoses for trees #0, #1, and #7 (“pred_0,” “pred_1,” and “pred_7”) could not be generated due to missing values.

Nine of those 14 cases that did not include the diagnosis only had the records of ID, evaluation number, and sex, as shown in Figure 8. Therefore, no classification could be obtained for those cases. The remaining five cases had missing values in some of their records that prevented obtaining a result in one or more trees. Figure 10 shows one of those cases, where trees #0, #1, and #7 were not activated. However, the result shown by the rest of the decision trees that make up the tree ensemble [encoded in the properties “pred_(*n*)”] would allow a final diagnosis of MCI for that case, by summing the classifications and then comparing it with the established threshold (*th* = 2). This way the ensemble manages the missing values, which focus on the data it has without altering the original database and allows a first insight into the cognitive state of the subjects. However, such classifications should be treated with caution, especially if the number of total activated trees is low and the result is under the threshold (which would give a preliminary and unsafe assessment of healthy).

This use case shows how the decision support system can be used for the evaluation of new cases obtained during cognitive screening. The system allows the classification of several hundred cases in a few minutes, even handling cases with missing data in one or more tests and obtaining the final classification from the activated decision trees.

#### 4.4.2 Incorporation of another database into the system

A significant objective of decision support systems is that their structure is functional in contexts other than those in which they are designed. To show how a different database can be coupled into this integrated system, we used a new anonymized database from the Dementia Disease Initiation (DDI) study (Fladby et al., 2017), a Norwegian MCI cohort composed of data collected across different medical centers and hospitals in Norway and focused on early detection of Alzheimer’s and other neurodegenerative dementias. This database integrates biomarkers, MRI, and neuropsychological tests. However, for this example, only *Classes* corresponding to raw scores on neuropsychological tests were selected. The sociodemographic data of the selected cases are detailed in Table 4.

**Table 4:** Summary of the Norwegian database.

Method	No. of subjects	Men/Women	Age mean (std)	Scholarity mean (std)	MMSE mean (std)
Healthy	447	209/238	64.79 (9.35)	13.74 (2.98)	29.09 (1.25)
MCI	387	180/207	66.20 (9.35)	13.60 (3.31)	27.26 (3.03)

First, the tests in both databases were analyzed for equivalences. Although the tests used in the Norwegian dataset encompassed similar objectives to those in the Spanish dataset, the divergence in test types, each with its own rules, scoring systems, and execution methods, hindered their classification as identical assessments. For example, the COWAT is a type of verbal fluency test but composed of different subtests than those used in the Spanish battery. The exception is the Trail Making Test A and B, being the same test in both databases. Therefore, it was necessary to repeat all the steps to adapt the system to the new database. First, the NIO ontology was reviewed to ensure that all tests presented in the Norwegian database were already modeled. Next, a new set of decision trees was generated, so they could establish a diagnosis from this new test battery. The same steps were followed as for the Spanish database: use of the bagging method for the generation of 11 decision trees, selection of the most efficient threshold, translation into SWRL rules, incorporation of these rules into the ontology, and evaluation of the performance of the system regarding six machine learning models (Table 5). The most efficient threshold was used to maximize *recall* for all models.

**Table 5:** Comparison between six machine learning models and the decision tree ensemble, using the threshold that maximize their performance for *F2*.

Method	F2	F1	Accuracy	Recall	Precision	ROC-AUC
ADAB *th* = 0.5	0.895	0.900	0.913	0.892	0.910	0.911
BAG *th* = 0.2	0.900	0.830	0.827	0.954	0.736	0.841
MLP *th* = 0.3	0.896	0.842	0.845	0.937	0.765	0.855
RLog *th* = 0.3	0.884	0.846	0.846	0.916	0.777	0.854
RF *th* = 0.2	0.881	0.766	0.766	0.961	0.663	0.787
SVM *th* = 0.3	0.894	0.856	0.856	0.926	0.787	0.863
Tree ensemble with *th* = 2	0.889	0.838	0.829	0.927	0.766	0.846

As in the Spanish database, the most efficient threshold was *th* = 2. As expected, the tree ensemble outperforms the individual tree average as in the Spanish database. In comparison with other ML systems, the tree ensemble performance is in the middle of the other systems, scoring the best *F2* the BAG with *F2* = 0.900, and the third method in having both the highest *F2* and *recall* (*F2_TE2_* = 0.889, *recall_TE2_* = 0.927) surpassed only by BAG (*F2_BAG_* = 0.900, *recall_BAG_* = 0.954) and MLP (*F2_MLP_* = 0.896, *recall_MLP_* = 0.937).

#### 4.4.3 Relationship between different domains modeled in the ontology

NIO is an ontology designed to model four different interrelated domains: neuropsychological tests, cognitive domains, brain areas, and neurodegenerative diseases. In this use case, we took advantage of it to relate low performance in verbal fluency tests with potential alterations in cognitive functions and brain areas associated with these tests. First, we searched the literature for which cognitive functions and brain areas related to each neuropsychological test (Baldo et al., 2006; Prescott et al., 2006), as well as the thresholds above which impairment is considered (García-Herranz et al., 2019). Next, the necessary relationships between the *Classes* and *Data Properties* involved were established through SWRL rules. Finally, the reasoner was activated to obtain the inference of those relationships, along with the “Healthy/MCI” classification.

**Figure 11:**
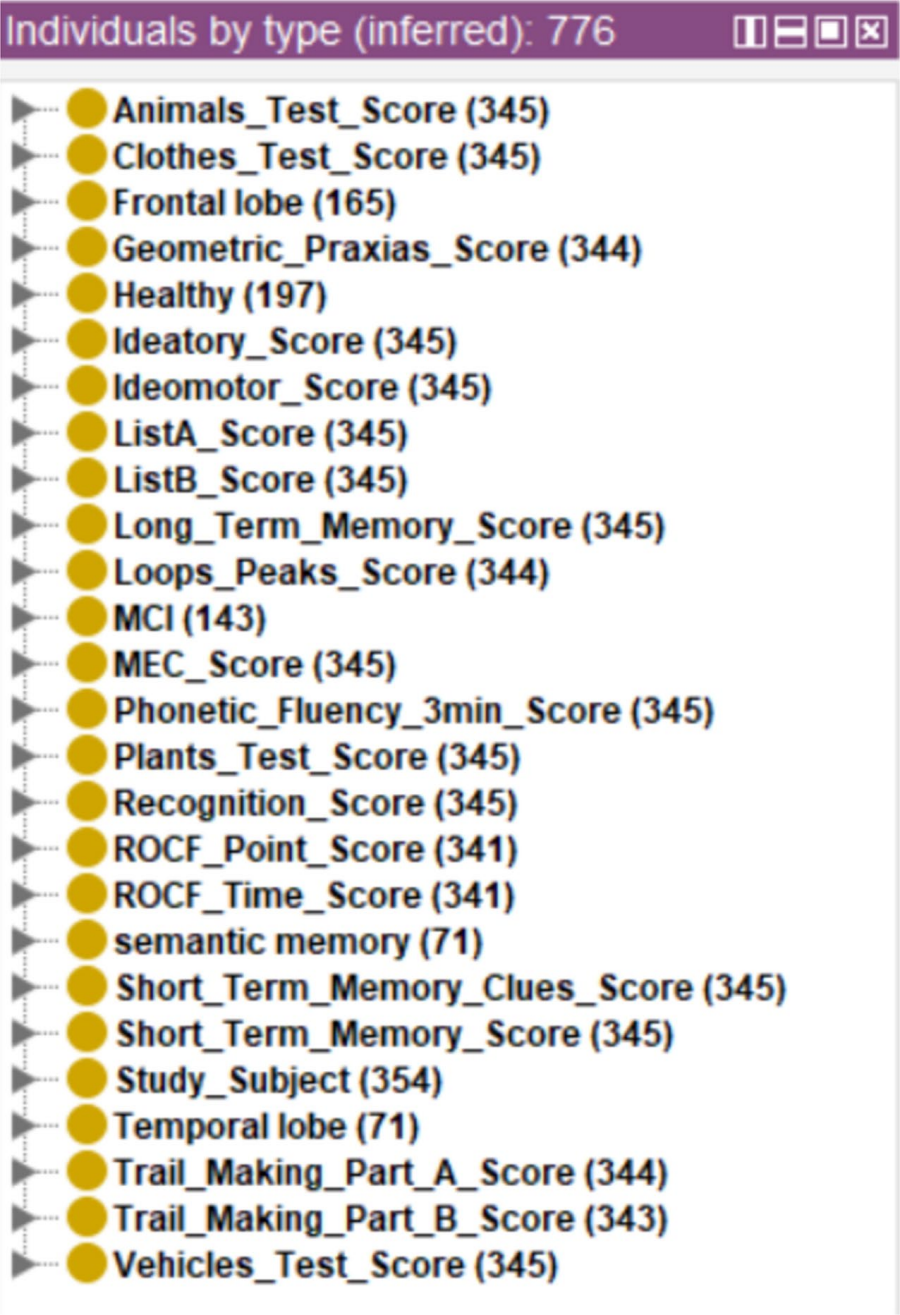
Inference of possible cognitive or brain alterations from the set of cases without diagnosis.

The results can be seen in Figure 11, where 71 cases were classified with possible temporal lobe damage, 165 with frontal lobe damage and 71 with possible semantic memory problems.

**Figure 12:**
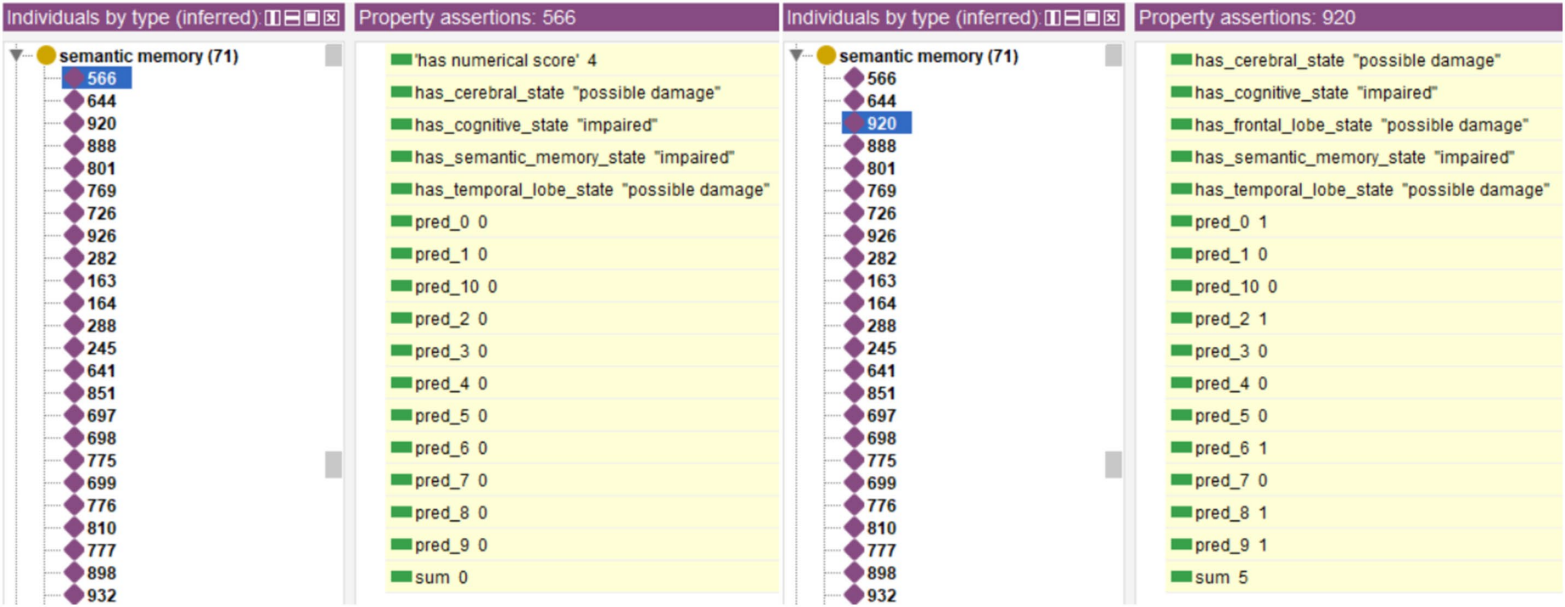
Example of two cases marked as having possible damage in the cognitive domain of semantic memory, with one of the cases evaluated as Healthy, and the other as MCI.

Figure 12 shows as an example two cases of *Individuals* who present potential alterations in semantic memory, one classified as “Healthy” and another as “MCI.” The system assigns the status of “impaired” to the associated *Data Property* of “has_semantic_memory_state,” and the status of “possible Damage” to the properties of “has_temporal_lobe_state” (both cases) and “has_frontal_lobe_state” in one of them (case 920).

These complementary relationships would allow a deeper understanding of the cognitive and physical status of the subjects, allowing the refinement of both follow-up and diagnosis. This would lead to a more accurate identification of the type of MCI of each subject and which disease it is most likely to lead to. This use case also shows that the relationship established between performance and cognitive functions/brain areas is independent of diagnosis, allowing a complementary analysis using the semantic relationship between different domains given by the ontology.

## 5 Discussion

Owing to the operational mechanisms of the SWRL rules, the system meticulously records all intermediary decisions involved in deducing the final diagnosis from data. Its integration in an ontology allows the recovery of the inference process followed by the reasoner to reach a specific diagnosis. Using a decision tree ensemble instead of a single tree increases the system power, making it more accurate at identifying cases with MCI and less prone to overfitting the training set. Furthermore, the integrated system allows cases with missing data to be treated directly, without the need for prior data preprocessing. This avoids the possible inclusion of artifacts in the system.

*Recall* measures the model’s ability to retrieve positive samples (Gupta et al., 2021). Therefore, for our study, it is of great interest to increase *recall* to detect as many positive cases as possible. However, using *recall* alone without considering *precision* may result in a trivial model that would classify all subjects as MCI by default, while the goal of screenings is to reduce the number of subjects to focus on. *F2* was selected as the most appropriate metric to evaluate the performance of a screening method as it gives more weight to *recall* while keeping a balance between *FP* and *FN*. Higher *F-score* was discarded as it could turn the screening system into a trivial one.

Two conclusions were drawn from the analysis of the tree ensemble thresholds. First, the most efficient threshold for a scenario in which both *recall* and *precision* would be optimized with 11 trees corresponds to *th* = 5. This means that a minimum of 5 decision trees would be necessary to issue a diagnosis of MCI and classify a case as such. Second, the tree ensemble with *th* = 2 is the most efficient detecting MCI cases, with *F2_TE2_* = 0.830 just after the BAG (*F2_BAG_* = 0.832), as shown in Table 3. Therefore, the performance of the system is suitable to identify MCI subjects. It is worth noting that, in general, the thresholds in all machine learning models were low (from *th* = 0.2 to *th* = 0.4). This could indicate that when MCI symptoms are still mild, a low threshold would help detect a higher number of MCI cases that otherwise would have been classified as healthy.

The following can be inferred from the three use cases shown. In the first one, the integrated system can issue a diagnosis of new cases fast and without the need for preprocessing that could alter the data. The system also allows physicians and neuropsychologists to review each case and the inference followed if necessary. It is also possible to establish a diagnosis in those cases with missing data, even though the final diagnosis could not be obtained, using the “partial diagnosis” of the activated trees. The use of the original records, without the need for prior preprocessing to remove or impute missing values before analysis, allows experts to focus on the evaluation and diagnosis of the subjects. This shows the system’s ability to be used as a population screening tool, saving time in the diagnostic evaluation and, therefore, allowing more people to be reached.

In the second use case, the use of another database revealed the great heterogeneity present in the field of early detection of Alzheimer’s disease through neuropsychological tests. Because almost none of the tests presented in the Spanish and Norwegian databases matched, it was necessary to first check that all tests were modeled in the ontology. It was also necessary to generate a new tree ensemble model able to establish a classification from the new database. However, the process of creating and evaluating the new tree ensemble was faster as the entire system methodology was already defined. The performance analysis showed that the system with threshold *th* = 2 was among the best models when considering both *F2* and *recall* for detecting MCI cases. Also, all methods present a clear improvement compared to the results with the Spanish database. Future analyses are needed to determine the reasons for the discrepancies in the performance of both databases.

The last use case showed how the ontology’s semantic relationship capabilities can be used to relate data from different domains. Specifically, to identify cognitive problems and potentially affected brain areas using their test performance. This complementary analysis can help to highlight the main cognitive problems of a subject regardless of his or her diagnosis. However, the information that links neuropsychological tests with both cognitive processes and brain areas is scarce, scattered, and often contradictory (Gomez-Valades et al., 2021), even in widely used and studied tests such as verbal fluency tests. Therefore, although an increase in this type of relationships may help to refine the MCI diagnosis, identifying the type of MCI and what kind of neurodegenerative disease is more likely to lead based on the test performance, in-depth modeling of this type of relationships is a project in its own, and it is left as future work.

### 5.1 System limitations

The system can make inferences on cases with missing data and issue diagnoses from the decision trees not affected by the missing data. However, because both OWL and SWRL rules assume an open world, the system is not able to establish an automatic final diagnosis in case missing values cause one or more of the rule sets corresponding to a decision tree fail to activate. In such cases, the diagnosis is obtained semi-automatically, where partial classification are obtained automatically(the results given by the SWRL rules of the activated decision trees), and the final diagnosis must be obtained manually by summing them and comparing the result with the established threshold. Of course, this classification should be treated with caution because it is based on partial information and it is only conclusive when the classification threshold is exceeded.

Finally, we are aware that the ML system’s performance is not optimal because most of the subjects with MCI were at a very early stage, presenting very mild symptoms that could be mistaken for normal aging, and the small size of the sample does not allow for the training of a robust and reliable ML model.

## 6 Conclusion

This paper presents the design of a decision support system that integrates an ontology with a tree ensemble written under SWRL. The system allows the explainability of the generated diagnosis while maintaining performance on par with other well-established ML systems. Its ontological base allows the system to operate within the ontological framework: integrating the data in the ontology allows the standardization and univocal interpretation of the stored data, and defining value limits for each test minimizes the inclusion of tests with erroneous values. The use of a small tree ensemble to obtain the diagnosis allows us to combine the explainability and translation capacity of the decision tree with the power of a bagging method. Integrating it within the ontology allows a reasoner to explain the reasoning process. The use cases show its practical utility in three additional contexts: direct cognitive screening from a dataset without requiring previous preprocessing, such as the one obtained during real population screenings which can have missing values; integrating the necessary rules so that the system can generate diagnoses from information in a different database; and establishing relationships between different domains based on the performance of subjects in the tests.

It demonstrates the ability of the system to be used to perform a preliminary automatic diagnosis of subjects, using the available results obtained in the neuropsychological tests. The system is designed to filter out as many suspected cases of MCI as possible, allowing its use as an initial screening method in primary care units for older adults. We also showed its ability to be extended with new knowledge, and to employ semantic capabilities for inference of new knowledge.

## Data Availability

The data analyzed in this study is subject to the following licenses/restrictions: The data used in this study is available for research purposes on reasonable request to M.R. Requests to access these datasets should be directed to Mariano Rincón, mrincon@dia.uned.es.

## References

[ref1] Baldo J. V. Schwartz S. Wilkins D. Dronkers N. F. (2006). Role of frontal versus temporal cortex in verbal fluency as revealed by voxel-based lesion symptom mapping. J. Int. Neuropsychol. Soc. 12, 896–900. doi: 10.1017/S1355617706061078, PMID: 17064451

[ref2] Clark D. G. McLaughlin P. M. Woo E. Hwang K. Hurtz S. Ramirez L.. (2016). Novel verbal fluency scores and structural brain imaging for prediction of cognitive outcome in mild cognitive impairment. Alzheimers Dement. Diagn. Assess. Dis. Monit. 2, 113–122. doi: 10.1016/j.dadm.2016.02.001, PMID: 27239542 PMC4879664

[ref3] Costa F. F. (2014). Big data in biomedicine. Drug Discov. Today 19, 433–440. doi: 10.1016/j.drudis.2013.10.012, PMID: 24183925

[ref4] Díaz-Mardomingo M. García-Herranz S. Rodríguez-Fernández R. Venero C. Peraita H. (2017). Problems in classifying mild cognitive impairment (MCI): one or multiple syndromes? Brain Sci. 7:111. doi: 10.3390/brainsci7090111, PMID: 28862676 PMC5615252

[ref5] Díaz-Mardomingo M. C. Peraita H. (2008). Detección precoz del deterioro cognitivo ligero de la tercera edad. Psicothema 20, 438–444, PMID: 18674440

[ref6] Fladby T. Pålhaugen L. Selnes P. Waterloo K. Bråthen G. Hessen E.. (2017). Detecting at-risk Alzheimer’s disease cases. J. Alzheimers Dis. 60, 97–105. doi: 10.3233/JAD-170231, PMID: 28826181 PMC5611830

[ref17] Fürnkranz J. Widmer G., (1994). Incremental reduced error pruning. In: Machine Learning Proceedings 1994. Elsevier, pp. 70–77.

[ref7] García-Herranz S. Díaz-Mardomingo M. C. Peraita H. (2016). Neuropsychological predictors of conversion to probable Alzheimer disease in ederly with mild cognitive impairment. J. Neuropsychol. 10, 239–255. doi: 10.1111/jnp.12067, PMID: 25809316

[ref8] García-Herranz S. Díaz-Mardomingo M. C. Venero C. Peraita H. (2019). Accuracy of verbal fluency tests in the discrimination of mild cognitive impairment and probable Alzheimer’s disease in older Spanish monolingual individuals. Neuropsychol. Dev. Cogn. B Aging Neuropsychol. 27, 826–840. doi: 10.1080/13825585.2019.1698710, PMID: 31822214

[ref9] Gomez-Valades A. Martinez-Tomas R. Rincon M. (2021). Integrative Base ontology for the research analysis of Alzheimer’s disease-related mild cognitive impairment. Front. Neuroinform. 15, –561691. doi: 10.3389/fninf.2021.561691, PMID: 33613222 PMC7889797

[ref10] Gomez-Valadés A. Martínez-Tomás R. Rincón-Zamorano M., (2019). Ontologies for early detection of the Alzheimer disease and other neurodegenerative diseases, in: Vicente J.M. Ferrández Álvarez-Sánchez J.R. Paz López F. de la Moreo J. Toledo Adeli H. (Eds.), Understanding the brain function and emotions, lecture notes in computer science. Vol. 11486. Switzerland: Springer Nature Switzerland AG, pp. 42–50.

[ref11] Gupta A. Anand A. Hasija Y., (2021). “Recall-based machine learning approach for early detection of cervical cancer,” in *2021 6th international conference for convergence in technology (I2CT)*. pp. 1–5.

[ref12] Ho T.K., (1995). “Random decision forests.” in *Proceedings of 3rd International Conference on Document Analysis and Recognition*. pp. 278–282, vol. 1.

[ref13] Hoehndorf R. Schofield P. N. Gkoutos G. V. (2015). The role of ontologies in biological and biomedical research: a functional perspective. Brief. Bioinform. 16, 1069–1080. doi: 10.1093/bib/bbv011, PMID: 25863278 PMC4652617

[ref14] Ivascu T. Manate B. Negru V., (2015). “A multi-agent architecture for ontology-based diagnosis of mental disorders.” in *2015 17th International Symposium on Symbolic and Numeric Algorithms for Scientific Computing (SYNASC)*. IEEE. pp. 423–430.

[ref15] Jensen M. Cox A. P. Chaudhry N. Ng M. Sule D. Duncan W.. (2013). The neurological disease ontology. J. Biomed. Semant. 4:42. doi: 10.1186/2041-1480-4-42, PMID: 24314207 PMC4028878

[ref16] Jitsuishi T. Yamaguchi A. (2022). Searching for optimal machine learning model to classify mild cognitive impairment (MCI) subtypes using multimodal MRI data. Sci. Rep. 12:4284. doi: 10.1038/s41598-022-08231-y, PMID: 35277565 PMC8917197

[ref18] Kang M. J. Kim S. Y. Na D. L. Kim B. C. Yang D. W. Kim E.-J.. (2019). Prediction of cognitive impairment via deep learning trained with multi-center neuropsychological test data. BMC Med. Inform. Decis. Mak. 19:231. doi: 10.1186/s12911-019-0974-x, PMID: 31752864 PMC6873409

[ref19] König A. Linz N. Tröger J. Wolters M. Alexandersson J. Robert P. (2018). Fully automatic speech-based analysis of the semantic verbal fluency task. Dement. Geriatr. Cogn. Disord. 45, 198–209. doi: 10.1159/000487852, PMID: 29886493

[ref20] Kotelnikov E. V. Milov V. R. (2018). Comparison of rule induction, decision trees and formal concept analysis approaches for classification. J. Phys. Conf. Ser. 1015:032068. doi: 10.1088/1742-6596/1015/3/032068

[ref21] Kulmanov M. Smaili F. Z. Gao X. Hoehndorf R. (2020). Machine learning with biomedical ontologies. Bioinformatics 36, 422–429. doi: 10.1093/bioinformatics/btz595, PMID: 31350877 PMC9883727

[ref22] Kulmanov M. Smaili F. Z. Gao X. Hoehndorf R. (2021). Semantic similarity and machine learning with ontologies. Brief. Bioinform. 22:bbaa199. doi: 10.1093/bib/bbaa199, PMID: 33049044 PMC8293838

[ref23] Lakshmi V.S. Nithya V. Sripriya K. Preethi C. Logeshwari K., (2019). “Prediction of diabetes patient stage using ontology based machine learning system.” in *2019 IEEE International Conference on System, Computation, Automation and Networking (ICSCAN)*. pp. 1–4.

[ref24] Linz N. Troger J. Alexandersson J. Konig A. (2017). Using neural word embeddings in the analysis of the clinical semantic verbal fluency task, International Conference on Computational Semantics, Computer Science. 7.

[ref25] Lobo A. Ezquerra J. Gómez Burgada F. Sala J. M. Seva Díaz A. (1979). Cognocitive mini-test (a simple practical test to detect intellectual changes in medical patients). Actas Luso Esp. Neurol. Psiquiatr. Cienc. Afines 7, 189–202, PMID: 474231

[ref26] López-de-Ipiña K. Martinez-de-Lizarduy U. Calvo P. M. Beitia B. García-Melero J. Fernández E.. (2018). On the analysis of speech and disfluencies for automatic detection of mild cognitive impairment. Neural Comput. & Applic. 32, 15761–15769. doi: 10.1007/s00521-018-3494-1

[ref27] Massari H. E. Gherabi N. Mhammedi S. Ghandi H. Qanouni F. Bahaj M. (2022a). An ontological model based on machine learning for predicting breast cancer. Int. J. Adv. Comput. Sci. Appl. 13:715. doi: 10.14569/IJACSA.2022.0130715

[ref28] Massari H. E. Gherabi N. Mhammedi S. Ghandi H. Qanouni F. Bahaj M. (2022b). Integration of ontology with machine learning to predict the presence of covid-19 based on symptoms. Bull. Electr. Eng. Inform. 11, 2805–2816. doi: 10.11591/eei.v11i5.4392

[ref29] Massari H. E. Sabouri Z. Mhammedi S. Gherabi N. (2022c). Diabetes prediction using machine learning algorithms and ontology. J. ICT Stand. 10, 319–338. doi: 10.13052/jicts2245-800X.10212

[ref30] Mežnar S. Bevec M. Lavrač N. Škrlj B. (2022). Ontology completion with graph-based machine learning: a comprehensive evaluation. Mach. Learn. Knowl. Extr. 4, 1107–1123. doi: 10.3390/make4040056

[ref31] Musen M. A. (2015). The protégé project: a look back and a look forward. AI Matters 1, 4–12. doi: 10.1145/2757001.2757003, PMID: 27239556 PMC4883684

[ref32] O’Connor M. Knublauch H. Tu S. Musen M., (2005). Writing rules for the semantic web using SWRL and Jess. Computer Science.

[ref33] Sherimon P. C. Sherimon V. Preethii S. P. Nair R. Mathew R. (2021). A systematic review of clinical decision support systems in Alzheimer’s disease domain. Int. J. Onl. Eng. 17, 74–90. doi: 10.3991/ijoe.v17i08.23643, PMID: 33091740

[ref34] Panza F. D’Introno A. Colacicco A. M. Capurso C. Del Parigi A. Caselli R. J.. (2005). Current epidemiology of mild cognitive impairment and other predementia syndromes. Am. J. Geriatr. Psychiatry 13, 633–644. doi: 10.1097/00019442-200508000-00002, PMID: 16085779

[ref35] Patrick J. Li M. (2012). An ontology for clinical questions about the contents of patient notes. J. Biomed. Inform. 45, 292–306. doi: 10.1016/j.jbi.2011.11.008, PMID: 22142949

[ref36] Pedregosa F. Varoquaux G. Gramfort A. Michel V. Thirion B. Grisel O.. (2011). Scikit-learn: machine learning in Python. J. Mach. Learn. Res. 12, 2825–2830. doi: 10.48550/arXiv.1201.0490

[ref37] Peraita H. García-Herranz S. Díaz-Mardomingo M. C. (2011). Evolution of specific cognitive subprofiles of mild cognitive impairment in a three-year longitudinal study. Curr. Aging Sci. 4, 171–182. doi: 10.2174/1874609811104020171, PMID: 21418005

[ref38] Petersen R. C. Aisen P. S. Beckett L. A. Donohue M. C. Gamst A. C. Harvey D. J.. (2010). Alzheimer’s disease neuroimaging initiative (ADNI). Neurology 74, 201–209. doi: 10.1212/WNL.0b013e3181cb3e25, PMID: 20042704 PMC2809036

[ref39] Petersen R. C. Caracciolo B. Brayne C. Gauthier S. Jelic V. Fratiglioni L. (2014). Mild cognitive impairment: a concept in evolution. J. Intern. Med. 275, 214–228. doi: 10.1111/joim.12190, PMID: 24605806 PMC3967548

[ref40] Prescott T. J. Newton L. D. Mir N. U. Woodruff P. W. R. Parks R. W. (2006). A new dissimilarity measure for finding semantic structure in category fluency data with implications for understanding memory organization in schizophrenia. Neuropsychology 20, 685–699. doi: 10.1037/0894-4105.20.6.685, PMID: 17100513

[ref41] Robinson P. N. Haendel M. A. (2020). Ontologies, knowledge representation, and machine learning for translational research: recent contributions. Yearb. Med. Inform. 29, 159–162. doi: 10.1055/s-0040-1701991, PMID: 32823310 PMC7442528

[ref42] Sahoo S. S. Kobow K. Zhang J. Buchhalter J. Dayyani M. Upadhyaya D. P.. (2022). Ontology-based feature engineering in machine learning workflows for heterogeneous epilepsy patient records. Sci. Rep. 12:19430. doi: 10.1038/s41598-022-23101-3, PMID: 36371527 PMC9653502

[ref43] Shoaip N. Barakat S. Elmogy M., (2019). “Alzheimer’s disease integrated ontology (ADIO).” in *2019 14th international conference on computer engineering and systems (ICCES)*. pp. 374–379.

[ref44] Shoaip N. Rezk A. El-Sappagh S. Abuhmed T. Barakat S. Elmogy M. (2021). Alzheimer’s disease diagnosis based on a semantic rule-based modeling and reasoning approach. Comput. Mater. Contin. 69, 3531–3548. doi: 10.32604/cmc.2021.019069

[ref45] Shoaip N. Rezk A. El-Sappagh S. Alarabi L. Barakat S. Elmogy M. (2020). A comprehensive fuzzy ontology-based decision support system for Alzheimer’s disease diagnosis. IEEE Access 9, 31350–31372. doi: 10.1109/ACCESS.2020.3048435

[ref46] Sirin E. Parsia B. Grau B. C. Kalyanpur A. Katz Y. (2007). Pellet: a practical OWL-DL reasoner. Web Semant. Sci. Serv. Agents World Wide Web 5, 51–53. doi: 10.1016/j.websem.2007.03.004

[ref47] Tsymbal A. Zillner S. Huber M. (2007). “Ontology – supported machine learning and decision support in biomedicine” in Data integration in the life sciences, lecture notes in computer science. eds. Cohen-Boulakia S. Tannen V. (Berlin Heidelberg: Springer), 156–171.

[ref48] Weakley A. Williams J. A. Schmitter-Edgecombe M. Cook D. J. (2015). Neuropsychological test selection for cognitive impairment classification: a machine learning approach. J. Clin. Exp. Neuropsychol. 37, 899–916. doi: 10.1080/13803395.2015.1067290, PMID: 26332171 PMC4809360

[ref49] Yesavage J. A. Brink T. L. Rose T. L. Lum O. Huang V. Adey M.. (1982). Development and validation of a geriatric depression screening scale: a preliminary report. J. Psychiatr. Res. 17, 37–49. doi: 10.1016/0022-3956(82)90033-4, PMID: 7183759

[ref50] Zekri F. Bouaziz R. Turki E., (2015). “A fuzzy-based ontology for Alzheimer’s disease decision support.” in *2015 IEEE International Conference on Fuzzy Systems (FUZZ-IEEE)*. IEEE, pp. 1–6.

[ref51] Zhang X. Hu B. Ma X. Moore P. Chen J. (2014). Ontology driven decision support for the diagnosis of mild cognitive impairment. Comput. Methods Prog. Biomed. 113, 781–791. doi: 10.1016/j.cmpb.2013.12.023, PMID: 24468160

